# Supplementary material for: Enhanced Delivery of Rose Bengal by Amino Acids Starvation and Exosomes Inhibition in Human Astrocytoma Cells to Potentiate Anticancer Photodynamic Therapy Effects
Source: Cells. 2022 Aug 11;11(16):2502. doi: 10.3390/cells11162502 (PMC9406355; doi:10.3390/cells11162502)
Supplement: Supplementary file 1 [file cells-11-02502-s001.zip › cells-1840385-supplementary.pdf]

SUPPLEMENTARY MATERIALS

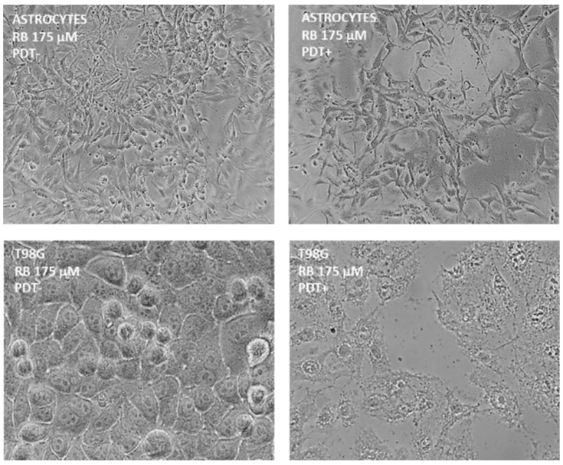

Figure S1. Optical microscope examinations following RB and PDT treatments. Normal rat primary astrocytes and T98G high grade astrocytoma cells were treated with RB (175 μM for 24 h) exposed (PDT+) or not- (PDT-) to 562/576 nm irradiation for 5 min and visualized using a Nikon Eclipse TS100 inverted microscope (20x magnification).

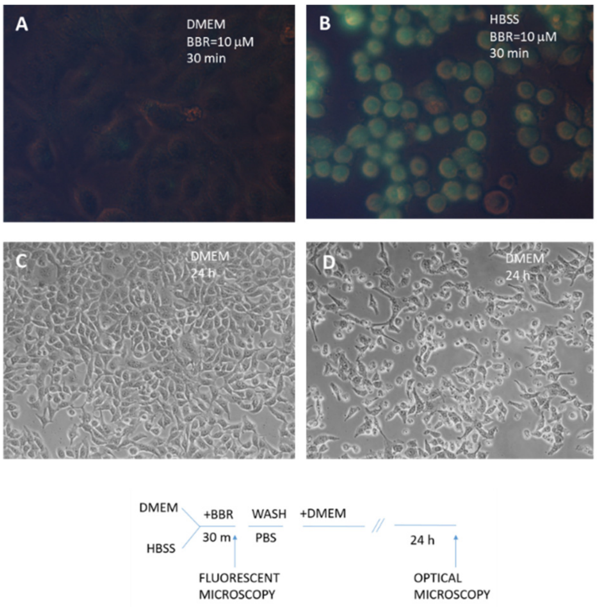

Figure S2. Fluorescent and optical microscope examinations following berberine (BBR) administration in normal growing conditions and amino acids starvation. (A,B) T98G cells were treated with BBR (10 μM) in standard DMEM high glucose (A) or in HBSS amino acids depleted media (B), both for 30 min and visualized using a Nikon Eclipse TS100 inverted fluorescent microscope (20x magnification, using UV2A blocking filter; exc.=355/50 nm; dichr. mirr.=400 nm; barrier=410 nm). (C,D) After washing with sterile PBS and DMEM medium replacement, not-starved (C) and previously amino acids starved (D) cells were observed, 24 h p.t., at 10x magnification by optical microscope. The experimental scheme of the treatments is reported below.

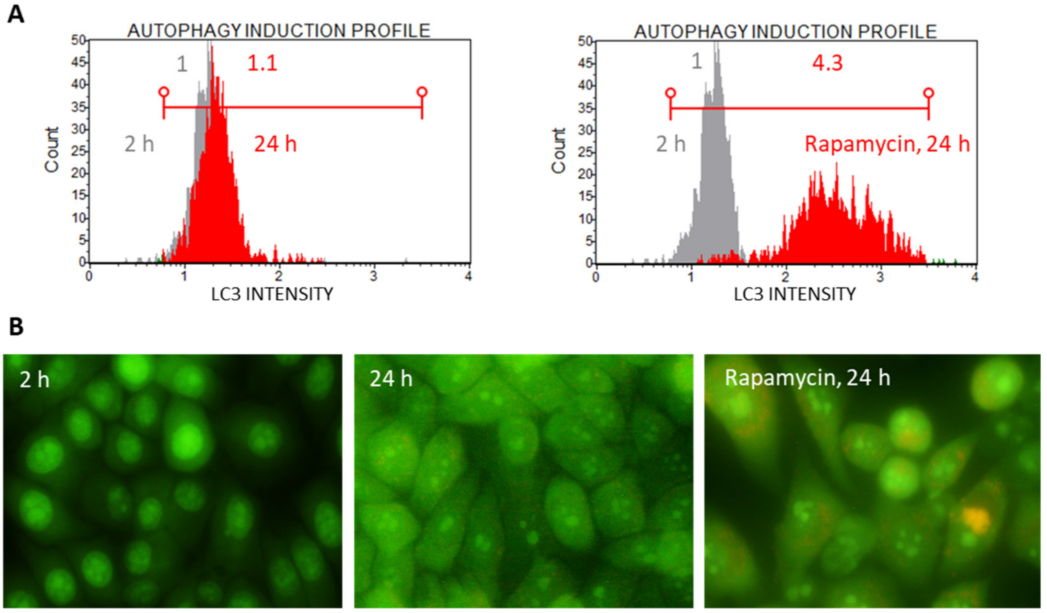

Figure S3. Autophagy evaluation in T98G cells in RB starvation to standard growing conditions. (A) LC3 cytofluorimetric evaluation using Guava Muse LC3-Antibody Based kit (Luminex) in T98G cells after RB (12.5 µM) starvation in HBSS (grey plot) and following 24 h at standard growing conditions (red) (left plot); LC3 expression following RB starvation (grey plot at right) was also compared to T98G cells treated for 24 h with Rapamycin (1 µM) (red plot). (B) Inverted fluorescent microscope examinations of T98G cells after RB (12.5 µM) starvation by HBSS (2 h) and following 24 h at standard growing conditions, compared to cells treated for 24 h with Rapamycin (1 µM); cells were stained for 5 min with Acridine orange dye (Sigma, 0.1 µg/ml), washed with PBS and visualized using a Nikon Eclipse TS100 inverted fluorescence at 40x magnification (B2A blocking filter; exc.=470/40 nm; dichr. mirr.=505 nm; barrier=510 nm).

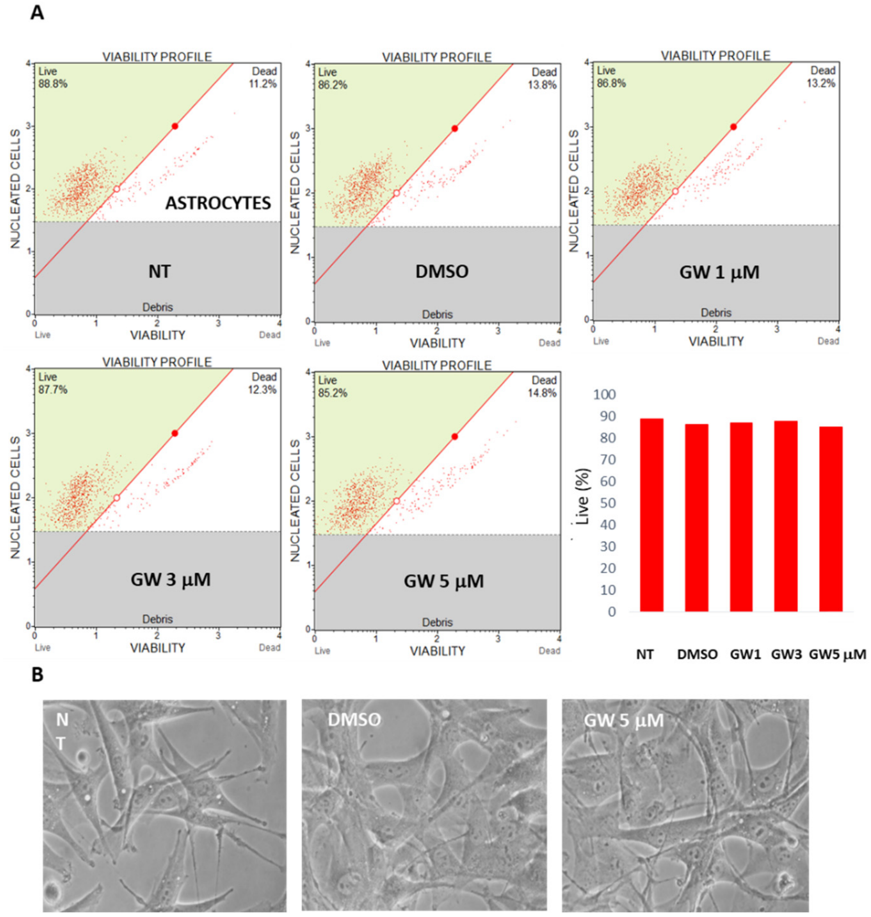

Figure S4. Effect of GW4869 on rat normal astrocytes viability. (A) Astrocytes were incubated with different GW4869 (GW) concentrations (i.e. 0-1-3-5 μM) and assayed in viability for 24 h in standard growing media (high glucose) using the cytofluorimetric Guava Muse Count & Viability kit. As a mock control of the possible solvent toxicity, DMSO was tested at the corresponding volume used to assay the highest GW4869 dose (GW=5 μM). (B) Optical microscope evidences at 24 h p.t. of untreated (NT), mock (DMSO) and GW=5 μM using a Nikon Eclipse TS100 inverted optical microscope at 40x magnification.

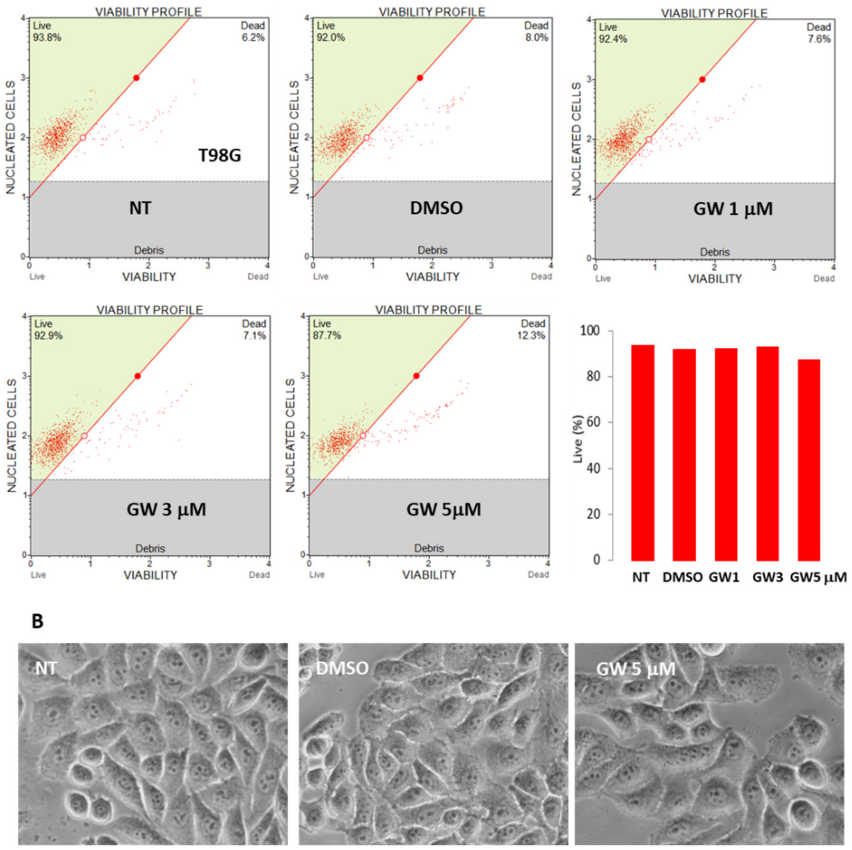

Figure S5. Effect of GW4869 on T98G cell viability. (A) T98G cells were incubated with different GW4869 (GW) concentrations (i.e. 0-1-3-5 μM) and assayed in viability for 24 h in standard growing media (high glucose) using the cytofluorimetric Guava Muse Count & Viability kit (Luminex). As a mock control of the possible solvent toxicity, DMSO was tested at the corresponding volume used to assay the highest GW dose (i.e. 5 μM). (B) Optical microscope evidences at 24 h p.t. of untreated (NT), mock (DMSO) and GW=5 μM using a Nikon Eclipse TS100 inverted optical microscope at 40x magnification.

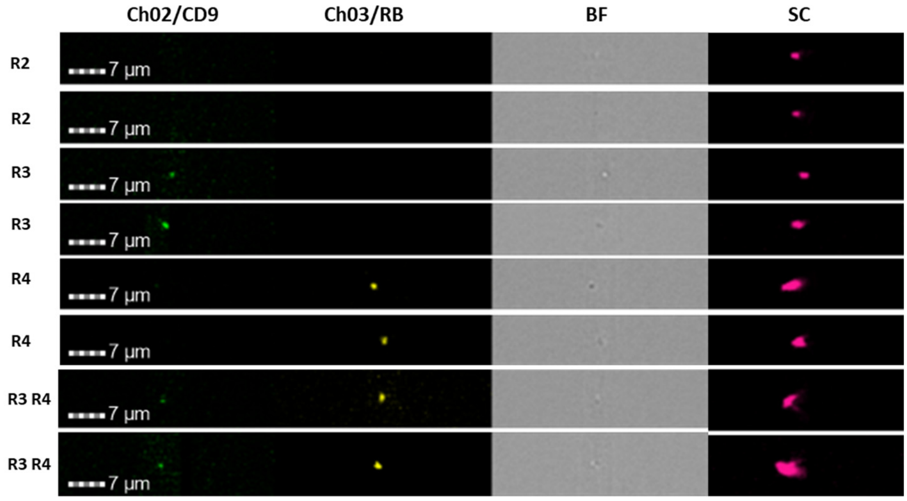

Figure S6. Flow cytometric characterization of exosomes. Additional image galleries related to the experiment described in Figure 7, according to the defined R gates. BF=bright field; SC=size scatter.

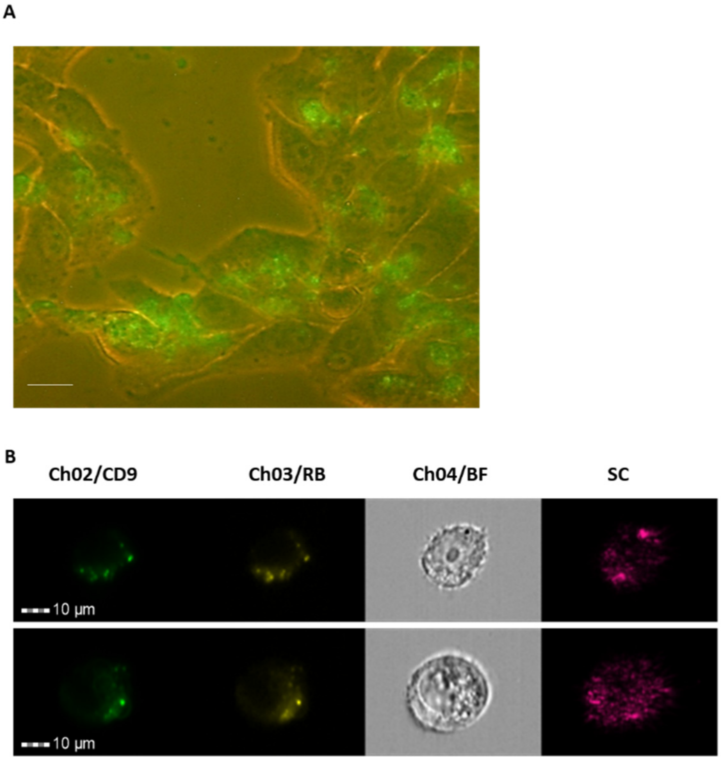

Figure S7. Cellular internalization of CD9+/RB+ exosomes. (A) Exosomes isolated from T98G cells, previously treated with RB (12.5 µM), stained with CD9-Dylight488 conjugated antibody and column purified from dyes excess (Exosome spin columns, MW 3000, Thermofisher), were administered to growing T98G cells and visualized using a Nikon Eclipse TS100 inverted fluorescence (B2A blocking filter; exc.=470/40 nm; dichr. mirr.=505 nm; barrier=510 nm) at 40x magnification. Scale bar= 10 µm. (B) CD9+/RB+ exosomes were also visualized in T98G cells by Amnis Imagestream flow cytometry (Luminex) at 60x magnification. BF=bright field; SC=size scatter.

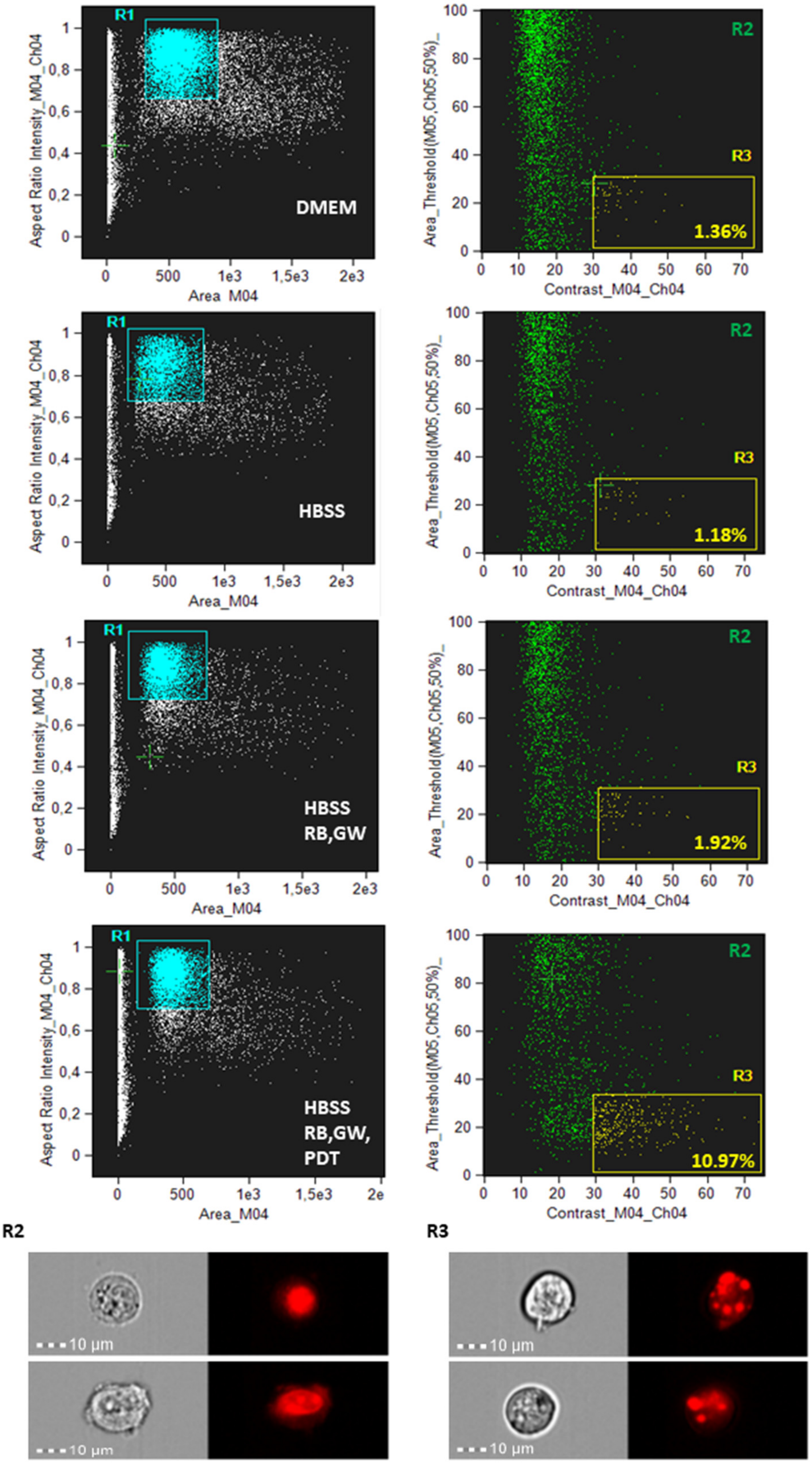

Figure S8. Nuclear fragmentation analysis. Amnis ImageStream flow cytometry analysis of nuclear fragmentation in T98G cells in standard growing (DMEM), in HBSS starvation alone (30 min), after RB (0.75 M) and GW (5 M) HBSS starvation (30 min), in absence or presence of PDT stimulation (2 min at 562/576 nm). Trypsinized cells were analyzed for bright field (Ch04) and for nuclear fluorescence (Ch05) using DRAQ5

dye (Invitrogen, 1/1,000 dilution for 15 min at 37°C) at 60x magnification. The IDEAS wizard “Apoptosis” was applied to focused cells (R1 gates) gating R3 as high contrast (Contrast_Mo4_Ch04) and low nuclear area (Area Threshold (M05, Ch0.50%) values. R2 and R3 gates indicated integrity and fragmented nuclei, respectively. Cell images representative of R2 and R3 galleries are reported.

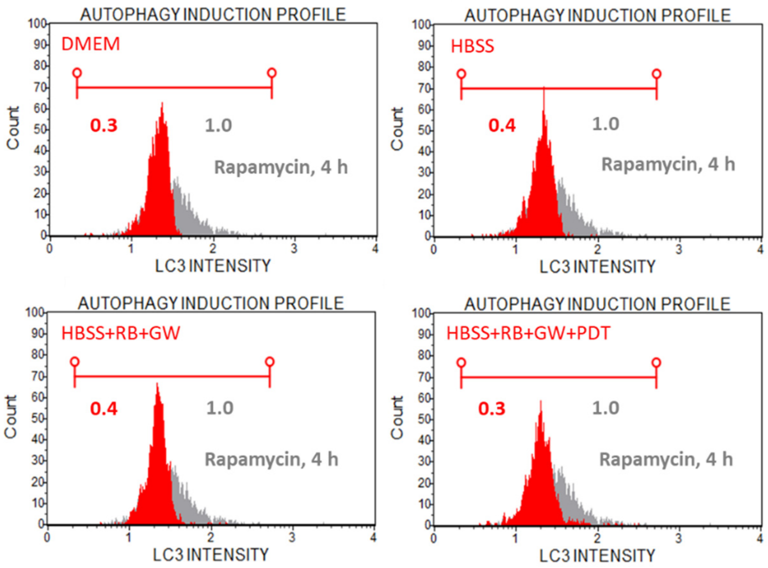

Figure S9. Autophagy flux evaluation in T98G cells. LC3 cytofluorimetric evaluation using Guava Muse LC3-Antibody Based kit (Luminex) in T98G cells in normal growing condition with high glucose (DMEM); after amino acids starvation (30 min) and following 24 h at standard growing conditions (HBSS); after amino acids starvation (30 min) in presence of RB (0.75 µM), after addition of GW4869 (5 µM) and following 24 h at standard growing conditions (HBSS+RB+GW); after amino acids starvation (30 min) in presence of RB (0.75 mM), after addition of GW4869 (5 mM), PDT exposition (2 min at 550 nm) and following 24 h at standard growing conditions (HBSS+RB+GW+PDT). LC3 expression after Rapamycin (1 µM for 4 h) was reported. All samples were treated, 4 h before trypsinization, with Autophagy Reagent A to prevent the lysosomal degradation of LC3 according to the manufacturer. LC3 intensity values were normalized to Rapamycin-treated sample (LC3=1.0, grey plots).
